# Genetic Diversity in *Passiflora* Species Assessed by Morphological and ITS Sequence Analysis

**DOI:** 10.1155/2014/598313

**Published:** 2014-06-22

**Authors:** Shiamala Devi Ramaiya, Japar Sidik Bujang, Muta Harah Zakaria

**Affiliations:** ^1^Department of Animal Science and Fisheries, Faculty of Agriculture and Food Sciences, Universiti Putra Malaysia Bintulu Sarawak Campus, 97008 Bintulu, Sarawak, Malaysia; ^2^Department of Aquaculture, Faculty of Agriculture, Universiti Putra Malaysia, 43400 Serdang, Selangor, Malaysia

## Abstract

This study used morphological characterization and phylogenetic analysis of the internal transcribed spacer (ITS) region of nuclear ribosomal DNA to investigate the phylogeny of *Passiflora* species. The samples were collected from various regions of East Malaysia, and discriminant function analysis based on linear combinations of morphological variables was used to classify the *Passiflora* species. The biplots generated five distinct groups discriminated by morphological variables. The group consisted of cultivars of *P. edulis* with high levels of genetic similarity; in contrast, *P. foetida* was highly divergent from other species in the morphological biplots. The final dataset of aligned sequences from nine studied *Passiflora* accessions and 30 other individuals obtained from GenBank database (NCBI) yielded one most parsimonious tree with two strongly supported clades. Maximum parsimony (MP) tree showed the phylogenetic relationships within this subgenus *Passiflora* support the classification at the series level. The constructed phylogenic tree also confirmed the divergence of *P. foetida* from all other species and the closeness of wild and cultivated species. The phylogenetic relationships were consistent with results of morphological assessments. The results of this study indicate that ITS region analysis represents a useful tool for evaluating genetic diversity in *Passiflora* at the species level.

## 1. Introduction

*Passiflora *is extensively grown in tropical and subtropical regions of the world. More than 500 species in this genus have been identified, and most of them are distributed throughout Central and South America [1]. Colombia is one of the main centers of* Passiflora* diversity which accommodates more than 100 species and is rich in nearly all sections of the genus [2].* Passiflora* species have been cultivated for their edible fruits, ornamental flowers, and pharmaceutical uses. The* Passiflora* genus contains the highest number of species in Passifloraceae with edible fruit [3]; in addition, the plants of this genus have desirable organoleptic properties [4].

At the early 20th century, two major technical monographs laid the modern foundation for* Passiflora* systematics, including works by Harms [5] and Killip [6]. The species were divided into 22 subgenera based on floral morphology by Killip [6]. Later, the infrageneric taxonomy of* Passiflora* has undergone significant revision based on morphological traits by Feuillet and MacDougal [7] and four subgenera were recognized:* Passiflora, Decaloba, Deidamioides, *and* Astrophea*. While progress has been made in understanding phylogenetic relationships of subgenus* Passiflora,* little is known about the relationship among them. The subgenus* Passiflora* is the largest in genus of* Passiflora* which comprised about 240 species generally regarded as typical passionflowers. Species of subgenus* Passiflora* are characterized by handsome flowers that are dominated by corona [1]. Feuillet and MacDougal [7] subdivide the representatives of subgenus* Passiflora* into six supersections and further divided into sections or series based on their morphological characteristics.

The species of the* Passiflora* genus have a wide range of morphological characteristics, anatomical differences, and phylogenetic variability. According to Sánchez et al. [8],* Passiflora *species are difficult to classify because some species vary widely in terms of morphology and other species closely resemble each other. Taxonomic classifications of* Passiflora* are typically based on morphological [9–11], ecological [12], and agronomic variations [13]. For example,* P. edulis* is generally characterized according to production, fruit weight, fruit size, juiciness, and juice acidity; however, these variables do not represent precise quantitative traits at the taxonomic level [9]. Moreover, existing inter- and intraspecies dissimilarity among the* Passiflora* species makes understanding the link between morphological plasticity, genotypic diversity, and speciation challenging. Increasing numbers of various interspecific hybrids have been produced in this genus [14], thus contributing to broad morphological variability.

Germplasm characterization based on molecular phylogeny can also contribute to a better understanding of the evolutionary process and genetic divergence of accessions. Assessing genetic diversity in* Passiflora* is also important for the utilization and conservation of the germplasm. The genetic background of the plant is a crucial factor for plant breeders when selecting the parental material for breeding [15, 16]. Over the years, studies have been carried out to examine the phylogenetic relationships within the genera. Studies have reported attempts to clearly demarcate the species of the* Passiflora *genus using restriction enzymes (cpDNA sites; Yockteng and Nadot [17]; Paikrao et al. [18]), amplified fragments length polymorphism markers (AFLP; Ortiz et al. [4]; Segura et al. [15]), microsatellite markers (SSR; Ortiz et al. [4]; Oliveira et al. [19]), and inter-simple sequence repeats (ISSR; dos Santos et al. [11]). However, the genetic diversity of* Passiflora *species is mostly estimated with random amplified polymorphic DNA (RAPD; Fajardo et al. [20]; Aukar et al. [21]; Crochemore et al. [22]; Cerqueira-Silva et al. [23]).

The internal transcribed spacer (ITS) region of the nuclear ribosomal 18S-5.8S-25S rDNA locus, which has been used in phylogenetic studies of many angiosperm families, has proven particularly useful for improving our understanding of interspecies relationships [24]. The two internal transcribed spacer DNA sequences have evolved rapidly and are therefore useful for comparing closely related taxa. In some genera, variations in ITS sequences have proven useful for studies at the species level [25]. The ITS region is also flanked by well-conserved rRNA genes that can be used to differentiate plant species [26] such as lentils [27], peanuts [28], maize [29], and seagrass [30]. The ITS regions have also been used to assess the phylogeny of the genus* Passiflora*. In particular, a recent study by Mäder et al. [31] used ITS sequences to evaluate the intraspecific variability of 23 species of* Passiflora*. The first molecular phylogenetic analysis of* Passiflora* using plastid regions and ITS sequences was conducted by Muschner et al. [32]; these investigators studied the 61 species composing the entire subgenera and identified three major clades (*Passiflora, Decaloba, *and* Astrophea*).

Although insights into* Passiflora* phylogeny at the subgeneric level have been gleaned, genetic information on and evidence for monophyletic groups below this level are limited [15, 33, 34]. At lower levels, the use of an arbitrary selection of morphological characteristics to delimit genera has yielded conflicting results. More information is needed to address evolutionary questions at the interspecies relationship in subgenus* Passiflora* and DNA studies using sequence data from the genome may more accurately define this phylogeny. Accessing molecular polymorphisms at the species level also contributes to the acquisition of knowledge that can be useful for the conservation of the diversity of* Passiflora*. Interest in this agronomically important crop (particularly in the* Passiflora* subgenus) has grown. Therefore, we studied morphological characteristics and phylogenetic relationships of species of this genus by evaluating the genetic diversity and comparing the ITS sequence data from plants of different origins. The knowledge generated by these genetic-based characterizations provides information on the parental selection for breeding works and also may contribute to various aspects of future biological and ecological studies.

## 2. Materials and Methods

### 2.1. Sample Collection and Morphological Analysis

Plant materials were obtained from various regions of East Malaysia as presented in Table 1. Plant parts from cultivated* Passiflora* accessions* P. edulis* producing purple fruits (PE1),* P. edulis* producing dark purple fruits (PE2),* P. incarnata, P. quadrangularis* and* P. maliformis* were collected from the fruit farm of Universiti Putra Malaysia Bintulu Sarawak Campus (UPMKB) Bintulu (N 03° 12.45′ and E 113° 4.68′), Sarawak. The five plant species were grown from seeds acquired from the commercial supplier Trade Winds Fruit, Windsor. In addition,* P. edulis* producing pink red fruits (PE3) and* P. edulis* producing yellow fruits (PE4) were collected from small-scale passion fruit farms at Kota Kinabalu (N 05° 58.28′ and E 116° 5.72′), Sabah and Ba'kelalan (N 03° 58.44′ and E 115° 37.08′), Sarawak, respectively. Wild* P. foetida* possessing flowers with green bracts (PF1) and* P. foetida* possessing flowers with red bracts (PF2) were collected from bushes at Bintulu (N 03° 10.25′ and E 113° 2.39′), Sarawak. Data on vegetative and reproductive morphology were recorded, and quantitative features were determined for the aril parts; leaves, stems, tendrils, bracts, flowers, fruits and seeds. Specimen identification and botanical nomenclature were based on the taxonomic keys of Ulmer and MacDougal [1].

### 2.2. Phylogenetic Analysis

#### 2.2.1. Polymerase Chain Reaction (PCR) Amplification

DNA was isolated from 0.01 g of fresh leaves using a modified alkaline lysis method without NaOH [35]. The nuclear ribosomal ITS regions were selected for PCR amplification and sequence analysis (forward (ITS1: TCCGTAGGTGAACCTGCGG) and reverse (ITS4: TCCTCCGCTTATTGATATGA)). The primers were chosen based on ITS sequences published by White et al. [36] and purchased in a lyophilized form from First BASE Laboratories, Malaysia. Each PCR reaction contained 13.0 *μ*L of sterile ultrapure water, 2.0 *μ*L of 10x NH_4_ PCR buffer, 0.4 *μ*L of 10 *μ*M dNTPs, 2.0 *μ*L of 50 *μ*M MgCl_2_, 1.0 *μ*L of 10 *μ*M forward and reverse primers, 1 unit DNA Taq polymerase, and 20–40 ng of template genomic DNA f8 or a final volume of 20.0 *μ*L. The PCR amplification of the ITS region was performed using an XP Thermal Cycler under the following conditions: one cycle of initial denaturation for 3 min at 95°C and 35 cycles of denaturation at 94°C for 30 s, annealing at 55°C for 30 s, and elongation for 1 min at 72°C. The final elongation step was conducted at 72°C for 5 min. The PCR products were quantified using 1% agarose gel electrophoresis; 6 *μ*L of PCR product were premixed with 6x loading buffer and loaded with 1 *μ*L of EZ-Vision Fluorescent Dye for the visualization of DNA bands in the agarose gel. The GeneRuler 1 kb plus DNA ladder was premixed with 6x loading buffer and used to measure the size of the obtained DNA fragments. Gel electrophoresis was run for 90 min at 90 V using a gel electrophoresis system. The gel was photographed using the AlphaView UV light imaging system. The excess dNTPs were removed from the amplified PCR products using the polyethylene glycol precipitation method [37], and the purified PCR products were sent to First Base Laboratory, Malaysia, for DNA sequencing. Both strands of the PCR product (reverse and forward) were sequenced using the Applied Biosystems BigDye Terminator v3.1 cycle sequencing kit.

### 2.3. Statistical Analysis

Data on morphological variables (leaf length, leaf width, stem width, tendril length, tendril width, bract length, bracts width, flower size, petal length, petal width, sepal length, sepal width, number of outer rows of corona, corona length, fruit weight, fruit length, fruit width, seed length, and seed width) were statistically analyzed using the SAS 9.0 for Windows. Single-factor analysis of variance (ANOVA) with post hoc Tukey's test (*P* ≤ 0.05) was used to compare the mean values. Discriminant analysis (DA) based on linear combinations of the predictor variables was used to find the maximum separation between the studied* Passiflora *species using XLSTAT 2013 for Windows.

The electropherograms of the sequence fragments were inspected and assembled using Phred, Phrap, and Consed software in MacPro [38]. The chromatograms were analyzed with Phred, assembled with Phrap, and scanned with PolyPhred; the results were then viewed with the Consed program. Only good-quality fragments (sequence quality above 20) were chosen for each sample. The nine studied* Passiflora* accessions sequences were compiled and aligned using multiple sequence comparison by log-expectation (MUSCLE) [39]. Other 30 additional in-group sequences were obtained from the GenBank database (NCBI) and included in the alignment (Table 1). Sites where gaps were required to maintain the alignment of the sequences were treated as missing data. As in an earlier analysis,* Mitostemma brevifilis* and* Paropsia madagascariensis *were chosen as outgroups [32].

Phylogenetic relationships were developed using maximum likelihood (ML) and maximum parsimony (MP) with the MEGA 5.1 software [40]. Maximum likelihood is a method that seeks the tree that makes the data most likely. It applies an explicit criterion—the log—likelihood to compare the various models of nucleotide substitution in the presence of a large number of short sequences. Maximum likelihood tries to infer an evolutionary tree by finding the tree which maximizes the probability of observing the data [41]. The ML analysis using the Tamura 3-parameter model with gamma distributions (T92+G) was selected as the best-fitting substitution model. The best-fitting substitution method was chosen based on the Akaike information criterion (AIC), the Bayesian information criterion (BIC), and ln⁡*L* criterion. The model with the lowest AIC and BIC scores and the highest ln⁡*L* was chosen to best describe the substitution pattern [41]. Likelihood analysis was performed by initially determining the transition : transversion ratio (ts : tv) that maximized the log-likelihood value; more specifically, the range of ts : tv values was plotted against the corresponding inferred log-likelihoods. The sequences were analyzed using a heuristic search with bootstrap analysis based on 1000 replicates of the dataset. The resulting trees were saved and used as starting trees for random addition following nearest neighbor intersection (NNI) branch swapping. Maximum parsimony is based on the assumption that the most likely tree is the one that requires the fewest number of changes to explain the nucleotide sequence data in the alignment. Instead of models option used in other models, MEGA uses MP search model option to implement parsimony. The basic premise of parsimony is that taxa share a common characteristic because they inherited that characteristic from common ancestors [41]. We used the heuristic search method with simple taxon addition and tree bisection reconnection (TBR). The choice of nodes for branch swapping in the resulting parsimonious model was informed by bootstrap analyses consisting of 1000 replications of the heuristic search. The MEGA 5.1 program was also used to construct the phylogenetic tree and estimate sequence divergence [40].

## 3. Results and Discussion

### 3.1. Morphological Variation and Discriminant Analysis (DA)

**Figure 1:**
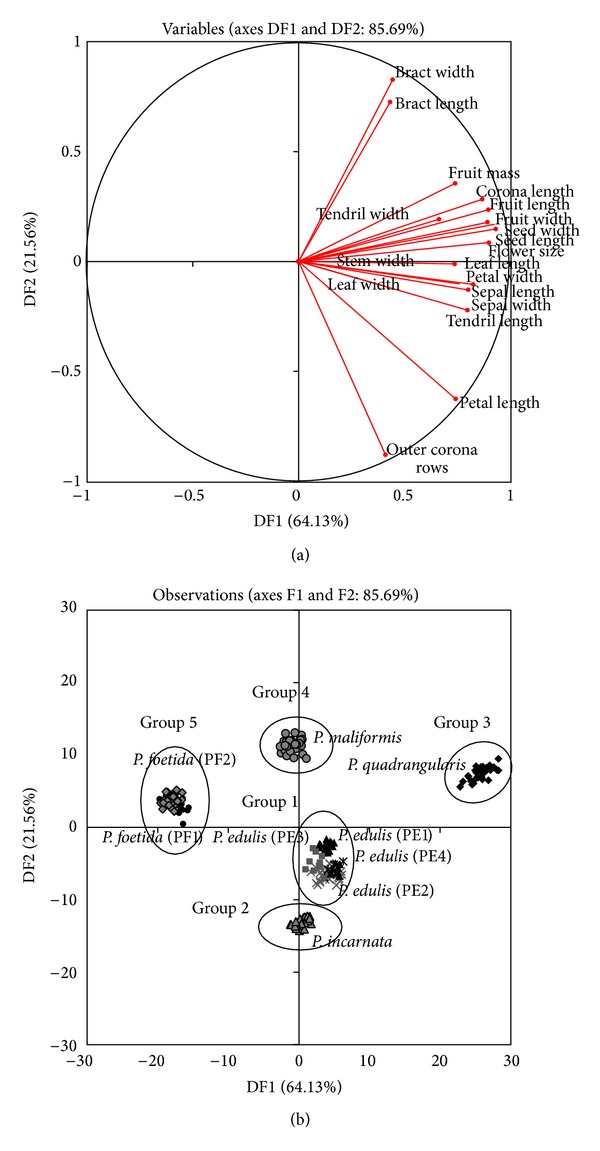
(a) Plot of the morphological parameters of the* Passiflora *accessions. Percentages in parentheses represent the variation in each component. (b) Positions of the DF scores of nine* Passiflora* accessions relative to DF1 and DF2.

Variations in the nineteen studied morphological characteristics of the* Passiflora* species are presented in Table 2. The vegetative and reproductive data were analyzed for discriminant analysis. Discriminant function analysis based on linear combinations of the variables produced better discrimination of the* Passiflora *species than the principal component analysis (data not shown). Biplots of the morphological dataset for discriminant functions (DF) one and two are shown in Figure 1. The discriminant function analysis that was based on linear combinations of the morphological variables accounted for 85.69% of the total variance (64.13% in DF1 and 21.56% in DF2). All of the morphological characteristics were loaded heavily on the positive ends of the plot.

We produced a scatter plot of 230 specimens for the first two discriminant functions based on morphological characteristics; the samples were densely arranged, and no overlapping characteristics were observed. The discriminant factors grouped the* Passiflora* species into five main clusters. The specimens belonging to Group 1 comprised cultivars of* P. edulis* (PE1, PE2, PE3, and PE4) were highly discriminated with respect to leaf, stem, sepal, and petal sizes. Accordingly, with the exception of fruit color and fruit sizes, we found no significant differences in morphological variables among cultivars of* P. edulis*. At ripening,* P. edulis* (PE1),* P. edulis* (PE2),* P. edulis* (PE3), and* P. edulis* (PE4) turn purple, dark purple, pink red, and yellow, respectively. The mean fruit sizes were also significantly different among cultivars. The fruit sampled from Ba'kelalan (PE4) was oval-shaped; consequently, the length of the fruit was statistically comparable to that of other* P. edulis* cultivars that produced round fruits. The fruits of* P. edulis* (PE3) (5.92 × 5.57 cm) and* P. edulis *(PE2) (5.48 × 4.68 cm) were smaller than those of* P. edulis* (PE1) (7.95 × 6.68 cm) and* P. edulis *(PE4) (9.02 × 6.49 cm). Group 2, which was composed of* P. incarnata* accessions, was related to the positive ends of the DF1 axis and the negative ends of the DF2 axis. The species of this group were also highly discriminated by number of outer corona rows; in addition, this cluster was closely related to the* P. edulis* cluster.

Group 3, consisting of* P. quadrangularis* accessions,was located at the right end of the DF1 axis, and the members of this group were highly discriminated by flower, fruit, and seed variables.* Passiflora quadrangularis* produced the largest flowers with longest coronas of all analyzed species; this species also produced the largest fruit (22.38 × 12.96 cm, with an average weight of 2.0 kg). Simultaneously, the size of the seed also was statistically different from all other species assessed. In all species except* P. quadrangularis*, the pulp represented 50–58% of the weight of the fruit; in* P. quadrangularis*, the pulp represented 11–15% of the fruit weight. The exocarp of the fruits of this group was thin, and the pulp was acidic, pale orange, and sweet. The ripened mesocarp was 2.5–3.0 cm thick which is edible.* Passiflora maliformis *was clearly separated into Group 4 near the positive ends of the DF2 axis, and the members of this group were highly correlated with respect to bract length and width. The bract structure of* P. maliformis *differed from that of other species; in particular, the three bracts of this species fused together and formed large cups around the bud or flowers. The bract of* P. maliformis *was twice as large (6.69 ± 0.16 cm) as that of* P. edulis* (3.37 ± 0.07 cm). The last group consisted of cultivars of* P. foetida: P. foetida *with green bracts (PF1) and* P. foetida *with red bracts (PF2). These species clearly diverged from the other cultivated* Passiflora* accessions that were located at the negative ends of the DF1 and DF2 axes. Few differences in plant morphology were observed between these two wild* Passiflora* cultivars with different bracts and fruit colors. The plant parts of both species were covered with numerous, small sticky glands and sticky hairs.

According to Martins et al. [42], the variation observed in fruit morphology is very common even at intraspecific level. The variation may be attributed to environmental factors or genetic differences or both. The variations in the fruit traits were attributed to differences in the age of the plant, fruit maturation stage, geographical sites, climatic condition, soil properties, and also seed origin [10]. Additionally, the complex nondominant inheritance of the external color of the fruit renders species identification even more challenging, as a number of intermediate colors can be produced in* P. edulis* [43]. In the present study, the cultivars of* P. edulis* were recognized and named accordingly with the International Code of Botanical Nomenclature based on their significant agronomic characteristics [43]. Assessments of morphological characteristics grouped the species according to their similarities. In accordance with the current study, Viana et al. [10] assessed the morphological diversity in six wild* Passiflora* species and stated the interspecific variability was observed for number of flowers, number of fruits, seeds, fruit length, fruit width, leaf area, and leaf length. Crochemore et al. [9] obtained clear separation among the 55 accessions consisting of 11 species from subgenus* Passiflora *by using morphological approach. The authors also recorded high divergence of* P. foetida* from other* Passiflora* probably due to the diversity of species studied and this was agreeable with Viana et al. [16] who worked with different accessions of cultivated and wild species of* Passiflora*.

### 3.2. Sequences Characteristics

The PCR products for all of the studied species were approximately 680 base pairs in length. The final dataset of aligned ITS sequence (including outgroup) used for the phylogenetic analysis consisted of 39 accessions from 13* Passiflora* species. The final alignment was highly variable, with 37.2% of sites that were parsimony informative. The guanine-cytosine (GC) content of the* Passiflora* species ranged from 60% to 64% and averaged 63%. Muschner et al. [32] reported similar GC contents in the* Passiflora* subgenus and stated that the GC content of* Passiflora* was higher than the GC content (53%) encountered in other subgenera (i.e.,* Decaloba, Adopogyne, Murucuja, Pseudomurucuja, *and* Deidamioides*). The genetic pairwise distance among taxa estimated with the Tamura 3-parameter model was calculated using complete sequence data (including data from outgroups); this distance ranged from 0 to 45.5% and averaged 16.1%.

### 3.3. Phylogenetic Analysis

Phylogenetic analyses were performed using maximum likelihood (ML) and maximum parsimony (MP) method. For ML analysis using the Tamura 3-parameter model with gamma distributions (T92+G) was selected as the best-fitting substitution model. The optimal range of ts : tv value was found to be 1.515 and this value was used in all subsequent maximum likelihood analyses. The obtained ML tree was constructed using a heuristic search that was performed using the NNI branch swapping option (−ln⁡*L* = 1426.54). For the MP analysis, the most parsimonious tree generated by phylogenetic analysis had a consistency index (CI) of 0.708, a retention index (RI) of 0.805, and a rescaled consistency index (RCI) of 0.569. The bootstrap analyses indicated high support for the main clades within the phylogenetic tree.

**Figure 2:**
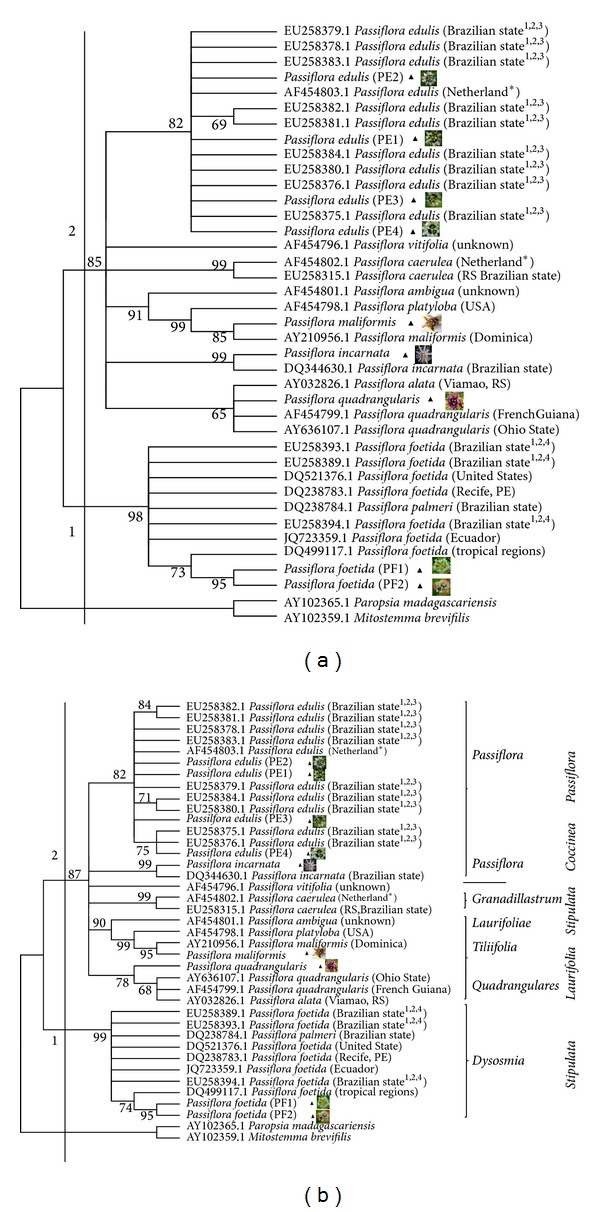
(a) Phylogenetic tree of* Passiflora* accessions inferred from the ML analysis using the Tamura three-parameter model. Only bootstrap scores greater than 60% are shown.* Mitostemma brevifilis* and* Paropsia madagascariensis *were used as outgroups. (b) Phylogenetic tree of* Passiflora* accessions inferred from parsimony analysis (full heuristic search with the tree bisection reconnection method).

The results of algorithms applied (ML and MP) showed that samples examined were distributed into two distinct well-supported clades. All of the* Passiflora* accessions occupied separate topological positions; that is, no overlap among species was observed. The resultant ML (Figure 2(a)) and MP trees (Figure 2(b)) were very similar; yet variation in the bootstrap support values and positioning of certain species was observed. Subgenus* Passiflora* was supported as monophyletic linkage in both ML and MP trees obtained. This is agreeable with finding of Muschner et al. [32] and Krosnick et al. [44]. The MP topology was chosen for discussion as it showed stronger bootstrap supports and gave insight into relationship within the subgenus* Passiflora *with* Passiflora* accessions examined arranged following their supersection and section or series recognized by Feuillet and MacDougal [7].

The MP analysis yielded a most parsimonious tree with high proportion informative sites (37.2%) and resulted in two well resolved major clades. As was observed with the ML analysis, a similar topological pattern was recorded for the cultivars of* P. foetida* in clade 1 (bootstrap score of 99%), which was basal to the clade containing other* Passiflora* accessions (i.e.,* P. caerulea, P. incarnata, P. maliformis, P. quadrangularis, P. vitifolia, P. alata, P. platyloba, *and* P. edulis*). The constructed phylogeny tree was in agreement with the morphological assessment, confirming the divergence of wild* P. foetida* from all other cultivated species. Clade 2 consisted of other* Passiflora* accessions with moderately strong bootstrap scores of 87%. In this clade, six well-separated subclades based on species similarities were formed. Subclade 1 consisted of all* P. edulis* individuals that clustered as a single group. The four accessions of* P. edulis* from East Malaysia were clustered in the same group and all of these accessions were well segregated based on their genetic similarity. In agreement with the ML analysis,* P. edulis *(PE4) occupied the base of this subclade. Subclade 2 consisted of* P. incarnata* accessions. Subclades 1 and 2 belonged to the* Passiflora* supersection.* Passiflora vitifolia,* which is a member of the Coccinea supersection, was clustered in subclade 3 and formed an independent lineage. Accessions of* P. caerulea* were clustered in subclade 4, with scores of 99%.* Passiflora caerulea* was assigned to categories based on morphological characteristics belonging to the Stipulata supersection and the Granadillastrum section, as proposed by Feuillet and MacDougal [7].* Passiflora foetida,* which belongs to the Stipulata supersection and the Dysosmia series, evolved separately from* P. caerulea*. A similar pattern was recorded by Muschner et al. [32], who observed high divergence between* P. caerulea *and* P. foetida*. Because of its divergence from other species in the* Passiflora* subgenus, the placement of Dysosmia into a separate subgenus, was proposed by Yockteng and Nadot [17] and is supported by the present finding as evident in Figure 2(b). Subclade 5 comprised* P. ambigua*,* P. platyloba, *and* P. maliformis,* and subclade 6 was composed of* P. quadrangularis* and* P. alata,* which both belong to the Laurifolia supersection. The* P. maliformis* and* P. platyloba *belonged to the same series (Tiliifolia), while* P. quadrangularis* was placed under series of Quadrangulares.

The present study revealed certain degree of variation detected in the ITS region sequence in all the individuals examined. This is in agreement with Mäder et al. [31], where in* Passiflora* the ITS region resulted in more informative sites than other markers (i.e., cpDNA). The ITS region provided greater resolution at the species level and was useful for differentiating the major groups of the* Passiflora *subgenus. Krosnick et al. [44] also have reported the ITS data provide greater resolution than ncpGS and trnL-trnF at the species level within* Passiflora*. Krosnick et al. [44] also showed that the subgenus* Passiflora* is monophyletic (97%) as observed in the present study compared to other subgenera (i.e., Deidamioides) which is polyphyletic.

Pattern of intraspecific variability in* Passiflora* using ITS region has been also studied by Muschner et al. [32] who investigate the relationship of 61 species of* Passiflora* composing the entire subgenera which was formally classified in 11 subgenera and representatives of four other genera. Based on the phylogenetic tree obtained from ML analysis yielded 3 major clades; representing subgenus* Passiflora*,* Decaloba* and* Astrophea* and the position of subgenus* Deidamioides* was undefined. The MP tree obtained was very similar to the respective ML tree was agrees with the current finding. The present results revealed some differences on the positioning of few* Passiflora* species in both the trees. Muschner et al. [32] stated that, although there are some consistent species grouping within the* Passiflora* clade, only few of them have high support and are consistent among markers and phylogenetic method, that is,* P. quadrangularis *and* P. alata* group. Most of this subgroup however, are not consistence with Killip's [6] subgenera or sections.

In accordance with the present finding, studies by Ossowski [45] revealed that within* Passiflora*, the group* P. menispermifolia, P. oerstedii, *and* P. caerulea* as well as the species pairs* P. platyloba* with* P. ambigua and P. quadrangularis* with* P. alata* are relatively well supported. The authors mentioned that the MP and ML trees obtained were not congruent and this was in contrast to the ML and MP topologies of present study. This contradicts the highly derived position in the parsimony tree. Mäder et al. [31] studied the intraspecific genetic diversity in 23 species of genus* Passiflora* consisting of subgenera* Decaloba* and* Passiflora* using ITS markers. The work by Mäder et al. [31] revealed that the* Passiflora *and* Decaloba *subgenera showed significant differences in the sizes of the ITS regions and in GC content, which can be related to reproductive characteristics of species in these subgenera. The clear seperation obtained within six species indicated that ITS may be a useful tool for the evaluation of intraspecific genetic variation in* Passiflora*.

The level of variation observed in the ITS region dataset was consistent with observations of Muschner et al. [32] and Krosnick and Freudenstein [46] and may be due to the percentage of missing sequence data for the four reference sequences used by Muschner et al. [32]; this variation complicated the final alignment using MUSCLE with different gap extensions. The final alignment was chosen based on the congruence in published relationships among outgroups [32, 46]. Besides the ITS region, other markers such RAPD, AFLP, SSR, and, cpDNA also make a direct comparison of these studies difficult and challenging; because of that intraspecific variation is not evenly distributed among species and led to different datasets for the same species (i.e.,* P. caerulea*,* P. edulis,* and* P. maliformis*). This may be attributed to the complexities of the evolutionary history of the genus and indicates that robust patterns would only emerge when different markers are considered together [31].

The molecular phylogenetic placement of the individuals in* Passiflora *subgenus were clearly separated than the subgenera of* Astrophea *or* Distephana *and was also supported by the morphological classification [45]. The present study showed, the MP phylogenetic tree was congruent with the classification based on morphological descriptions of Feuillet and MacDougal [7] where the subgenus* Passiflora* are categorized into six supersections. Morphologically, supersection* Passiflora* comprises those species that have serrate leaves, free serrate bracts, and upright flowers with a dominating corona as observed in* P. edulis* and* P. incarnata*. The supersection Stipulata is divided into three sections and section Granadillastrum with the richest species comprised plant with entire 3–5 lobed leaves and conspicuous upright flowers with free bracts (i.e.,* P. caerulea*). Essential characteristics of the species from supersection Laurifolia are large pendent flowers with predominant corona that surrounds the ovary in a campanulate fashion. This group possessed 3 series. When the bracts are connate, at least at base, this character is sufficient to assign the species to series Tiliifolia as recorded in* P. maliformis*. The* P. quadrangularis* is well known species of series Quadrangulares because of its winged, angled stems and possesses large, unlobed leaves with entire margin. Thus, the classification based on the morphological characteristics supports our phylogenetic topology in subgenus* Passiflora*. The present work provides the better integration of morphological data and ITS sequences to understand the relationship within subgenus* Passiflora*. Although our analyses considered only nine species, our results can be used to study phylogenetic relationships of closely related species and the separate topologies of different species.

## 4. Conclusion

This study provides an overview of a variety of genetically different individuals that could be commercialized in Malaysia and used in future breeding programs. Our results confirm that the ITS region provides high resolution at the species level and is useful for differentiating the major groups of the* Passiflora* subgenus. Although the analysis presented here was based on a limited number of species, the ITS region provided good resolution. Further studies using more species should be undertaken to obtain a good understanding of the evolutionary history of this plant at the species level and to enable the conservation of this economically important crop.

## Figures and Tables

**Table 1:** List of *Passiflora* accessions examined and individuals included in molecular analysis with their geographical locations, GenBank accession numbers, and references.

*Passiflora *accessions	Geographical locations	GenBank accession number	Citation
*Passiflora edulis* (PE1)	Bintulu, Sarawak, and Malaysia	Present sequence	Present study
*P. edulis* (PE2)	Bintulu, Sarawak, and Malaysia	Present sequence	Present study
*P. edulis *(PE3)	Kota Kinabalu, Sabah, and Malaysia	Present sequence	Present study
*P. edulis *(PE4)	Ba'kelalan, Sarawak, and Malaysia	Present sequence	Present study
*P. quadrangularis *	Bintulu, Sarawak, and Malaysia	Present sequence	Present study
*P. incarnata *	Bintulu, Sarawak, and Malaysia	Present sequence	Present study
*P. maliformis *	Bintulu, Sarawak, and Malaysia	Present sequence	Present study
*P. foetida* (PF1)	Bintulu, Sarawak, and Malaysia	Present sequence	Present study
*P. foetida *(PF2)	Bintulu, Sarawak, and Malaysia	Present sequence	Present study
*P. edulis *	Brazilian state^1,2,3^	EU258382.1	Mäder et al. [31]
*P. edulis *	Brazilian state^1,2,3^	EU258381.1	Mäder et al. [31]
*P. edulis *	Brazilian state^1,2,3^	EU258378.1	Mäder et al. [31]
*P. edulis *	Brazilian state^1,2,3^	EU258383.1	Mäder et al. [31]
*P. edulis *	Netherland	AF454803.1	Ossowski [45]
*P. edulis *	Brazilian state^1,2,3^	EU258379.1	Mäder et al. [31]
*P. edulis *	Brazilian state^1,2,3^	EU258384.1	Mäder et al. [31]
*P. edulis *	Brazilian state^1,2,3^	EU258380.1	Mäder et al. [31]
*P. edulis *	Brazilian state^1,2,3^	EU258375.1	Mäder et al. [31]
*P. edulis *	Brazilian state^1,2,3^	EU258376.1	Mäder et al. [31]
*P. quadrangularis *	French Guyana	AF454799.1	Ossowski [45]
*P. quadrangularis *	Ohio state	AY636107.1	Krosnick and Freudenstein [46]
*P. alata *	Viamao, RS	AY032826.1	Muschner et al. [32]
*P. incarnata *	Brazilian state	DQ344630.1	Muschner et al. [14]
*P. vitifolia *	Unknown	AF454796.1	Ossowski [45]
*P. caerulea *	Netherland	AF454802.1	Ossowski [45]
*P. caerulea *	Brazilian state^1^	EU258315.1	Mäder et al. [31]
*P. ambigua *	Unknown	AF454801.1	Ossowski [45]
*P. platyloba *	Horticultural, USA	AF454798.1	Ossowski [45]
*P. maliformis *	Dominica	AY210956.1	Muschner et al. [32]
*P. foetida *	Brazilian state^1,2,4^	EU258389.1	Mäder et al. [31]
*P. foetida *	Brazilian state^1,2,4^	EU258393.1	Mäder et al. [31]
*P. palmeri *	Brazilian state^1,2,4^	DQ238784.1	Muschner et al. [14]
*P. foetida *	United States	DQ521376.1	Hearn [47]
*P. foetida *	Brazilian state	DQ238783.1	Muschner et al. [14]
*P. foetida *	Ecuador	JQ723359.1	Thulin et al. [48]
*P. foetida *	Brazilian state^1,2,4^	EU258394.1	Mäder et al. [31]
*P. foetida *	Tropical regions	DQ499117.1	Wright et al. [49]
*Mitostemma* brevifilis*	Campo Grande^5^	AY102359.1	Muschner et al. [32]
*Pa* *ro* *ps* *ia* madagascariensis*	M Zyhra 949, WIS	AY102365.1	Muschner et al. [32]

Samples collected from various locations of Brazilian states: RS^1^: Rio Grande do Sul, SC^2^: Santa Catarina, MG^3^: Minas Gerais, PB^4^: Pernambuco, and MS^5^: Mato Grosso do Sul; tropical samples collected from New Guinea, northeast Australia, Borneo, India, Tahiti, and South America. ∗Outgroups species.

**Table 2:** Morphological parameters of nine *Passiflora* accessions.

Parameter	*Passiflora* species								
	*P. edulis* (PE1)	*P. edulis*(PE2)	*P. edulis* (PE3)	*P. edulis* (PE4)	*P. maliformis *	*P. quadrangularis *	*P. incarnata *	*P. foetida* (PF1)	*P. foetida* (PF2)
Vegetative morphology									
LL	13.82 ± 0.69^a^	13.71 ± 0.91^a^	13.12 ± 0.37^a^	13.85 ± 1.04^a^	12.03 ± 0.49^a^	13.66 ± 0.60^a^	8.71 ± 0.48^b^	7.34 ± 0.89^b^	6.42 ± 1.04^b^
LW	14.12 ± 0.16^a^	14.08 ± 0.36^a^	14.26 ± 0.92^a^	14.67 ± 0.52^a^	9.93 ± 0.69^b^	10.42 ± 0.42^b^	6.57 ± 0.57^d^	8.21 ± 0.71^c^	7.27 ± 0.86^cd^
STW	0.59 ± 0.54^ab^	0.56 ± 0.71^ab^	0.58 ± 0.34^ab^	0.57 ± 0.51^a^	0.49 ± 0.92^bc^	0.63 ± 1.08^ab^	0.39 ± 0.75^cd^	0.32 ± 0.47^d^	0.25 ± 0.26^d^
TL	24.43 ± 2.06^a^	26.95 ± 3.65^a^	25.83 ± 0.63^a^	23.56 ± 0.35^a^	23.87 ± 1.51^a^	26.63 ± 0.27^a^	23.52 ± 1.15^a^	16.03 ± 1.23^b^	13.38 ± 1.47^b^
TW	0.13 ± 0.05^a^	0.14 ± 0.03^a^	0.12 ± 0.01^a^	0.13 ± 0.04^a^	0.14 ± 0.02^a^	0.15 ± 0.01^a^	0.07 ± 0.06^b^	0.06 ± 0.02^b^	0.06 ± 0.02^b^

Reproductive morphology									
BL	3.37 ± 0.07^bc^	2.89 ± 0.07^bcd^	3.30 ± 0.12^bc^	3.46 ± 0.20^b^	6.69 ± 0.16^a^	2.59 ± 0.12^d^	2.32 ± 0.27^d^	2.73 ± 0.49^cd^	2.73 ± 0.18^cd^
BW	2.15 ± 0.02^b^	1.73 ± 0.04^bc^	2.13 ± 0.02^b^	2.49 ± 0.49^b^	4.23 ± 0.05^a^	1.22 ± 0.03^c^	1.28 ± 0.05^c^	2.32 ± 0.57^b^	2.35 ± 0.37^b^
FLS	8.91 ± 0.41^cd^	9.18 ± 0.58^c^	8.77 ± 0.23^cd^	9.09 ± 0.37^c^	10.54 ± 0.87^b^	12.16 ± 0.96^a^	8.37 ± 0.32^d^	4.20 ± 0.44^e^	3.86 ± 0.25^e^
PTL	3.64 ± 0.08^b^	3.89 ± 0.18^ab^	3.63 ± 0.09^b^	3.66 ± 0.08^b^	1.98 ± 0.30^c^	4.13 ± 0.16^a^	4.04 ± 0.07^a^	2.16 ± 0.26^c^	2.05 ± 0.14^c^
PTW	1.23 ± 0.04^bc^	1.15 ± 0.07^c^	1.37 ± 0.33^b^	1.22 ± 0.04^bc^	0.41 ± 0.02^e^	1.97 ± 0.03^a^	0.83 ± 0.04^d^	0.77 ± 0.03^d^	0.72 ± 0.04^d^
SPL	3.59 ± 0.08^b^	3.57 ± 0.09^b^	3.52 ± 0.11^b^	3.55 ± 0.13^b^	4.06 ± 0.12^a^	4.16 ± 0.11^a^	4.15 ± 0.09^a^	2.49 ± 0.15^c^	2.33 ± 0.20^c^
SPW	1.27 ± 0.11^b^	1.19 ± 0.31^b^	1.34 ± 0.45^b^	1.37 ± 0.38^b^	1.77 ± 0.16^a^	1.85 ± 0.19^a^	1.22 ± 0.10^b^	0.62 ± 0.03^d^	0.61 ± 0.04^d^
OCR	3.40 ± 0.52^bc^	3.70 ± 0.48^b^	3.70 ± 0.48^b^	4.00 ± 0.48^b^	2.00 ± 0.00^d^	3.00 ± 0.00^c^	5.00 ± 0.00^a^	2.00 ± 0.00^d^	2.00 ± 0.00^d^
CL	2.45 ± 0.40^c^	2.72 ± 0.18^c^	2.56 ± 0.16^c^	2.53 ± 0.26^c^	3.79 ± 0.85^b^	6.26 ± 0.50^a^	3.42 ± 0.47^b^	1.32 ± 0.22^d^	1.29 ± 0.26^d^
FM	83.3 ± 14.55^b^	49.8 ± 13.35^b^	64.4 ± 10.91^b^	124.4 ± 4.55^b^	35.5 ± 6.12^b^	2175.0 ± 51.57^a^	2.1 ± 0.66^b^	1.34 ± 7.45^b^	1.45 ± 6.19^b^
FL	7.95 ± 1.39^bc^	5.48 ± 0.89^cd^	5.92 ± 0.67^cd^	9.02 ± 0.77^b^	4.30 ± 0.57^d^	22.38 ± 2.53^a^	2.80 ± 0.03^de^	1.38 ± 0.23^e^	1.43 ± 0.35^e^
FD	6.68 ± 0.83^b^	4.68 ± 1.36^d^	5.57 ± 0.42^cd^	6.49 ± 0.37^bc^	3.88 ± 0.14^e^	12.96 ± 0.70^a^	2.45 ± 0.10^f^	1.44 ± 0.14^fg^	1.38 ± 0.11^g^
SL	0.63 ± 0.03^b^	0.61 ± 0.03^b^	0.59 ± 0.05^b^	0.63 ± 0.02^b^	0.61 ± 0.03^b^	0.98 ± 0.14^a^	0.56 ± 0.05^bc^	0.43 ± 0.03^cd^	0.41 ± 0.04^d^
SW	0.41 ± 0.02^b^	0.40 ± 0.02^b^	0.40 ± 0.01^b^	0.45 ± 0.02^b^	0.38 ± 0.03^b^	0.74 ± 0.06^a^	0.42 ± 0.05^b^	0.28 ± 0.01^c^	0.29 ± 0.02^c^

Different superscript letters within the same row indicate significant differences (Tukey's test, *P* ≤ 0.05) among the means of each variable of the *Passiflora *accessions. LL-leaf length (cm), LW-leaf width (cm), STW-stem width (cm), TL-tendril length (cm), TW-tendril width (cm), BL-bract length (cm), BW-bract width (cm), FLS-flower size (cm), PTL-petal length (cm), PTW-petal width (cm), SPL-sepal length (cm), SPW-sepal width (cm), ORC-outer corona rows, CL-corona length (cm), FM-fruit mass (g), FL-fruit length (cm), FD-fruit diameter (cm), SL-seed length (cm), and SW-seed width (cm).
